# Healthcare costs at the end-of-life among immigrant and non-immigrant groups in Manitoba, Canada

**DOI:** 10.23889/ijpds.v9i5.2621

**Published:** 2024-09-18

**Authors:** Charlotte Ma, Gangamma Kalappa, Nan Wang, Mahmoud Azimaee, Winnie Shen

**Affiliations:** 1 ICES

## Background

ICES has conducted Probabilistic Record Linkage (PRL) for heath and non-health data for over 15 years. The PRL process has made data linkable to many ICES data holdings. However, due to the manual gray area resolution, record linkages for large datasets are time consuming with inconsistent results.

## Approach

Adapting from many years of experience with PRL methodology, record linkage templates were built in SAS Dataflux. By using combination of deterministic linkage and fuzzy match algorithms, the multi-pass linkage strategy is maintained by passing unlinked records in subsequent linkage comparison cycles. The new template uses personal identifiers of multiple given names, surnames, date of birth, death date (if applicable) and sex. In each pass, the pool of possible matched pairs is created by merging on low sensitive matchcodes with different conditions. Then, using a rule-based approach, possible pairs are examined, flags are assigned, and survival rules are applied to select the best matched records.

## Results

By feeding a pre-prepared linkage file along with updating the input and output file names, the record linkage job will be conducted automatically in SAS Dataflux through submitting a “run” command. It significantly reduces the turnaround time with no clerical review required, achieves a similar linkage rate as PRL consistently, and is utilized by various research projects.

## Conclusion

The automated SAS Dataflux record linkage template is more efficient than the traditional PRL process. It eliminates the human intervention of gray area resolution, while reducing project turnaround times, and maintaining a comparable accuracy and linkage rate.

